# Evaluation of an automated template-based treatment planning system for radiotherapy of anal, rectal and prostate cancer

**DOI:** 10.1016/j.tipsro.2022.04.001

**Published:** 2022-04-12

**Authors:** Lucie Calmels, Patrik Sibolt, Lina M. Åström, Eva Serup-Hansen, Henriette Lindberg, Anna-Lene Fromm, Gitte Persson, David Sjöström, Poul Geertsen, Claus P. Behrens

**Affiliations:** a Department of Oncology, Copenhagen University Hospital - Herlev and Gentofte, Copenhagen, Denmark; b Department of Health Technology, Technical University of Denmark, Roskilde, Denmark; c Department of Clinical Oncology, Copenhagen University, Copenhagen, Denmark

**Keywords:** Template-based Ethos TPS, Online adaptive radiotherapy, Intelligent optimization engine, Automated treatment planning, Plan quality, Pelvic cancer, **AP**, automatically generated plan, **aTPS**, automated treatment planning system, **CN**, conformity number, **CT**, computed tomography, **CTV**, clinical target volume, **DVH**, dose volume histogram, **FFF**, flattening filter free, **GTV**, gross tumor volume, **HI**, homogeneity index, **IMRT**, intensity modulated radiotherapy, **KPB**, knowledge-based planning, **Linac**, Linear accelerators, **MCO**, multi-criteria optimization, **MLC**, multileaf collimator, **MP**, manually-generated plan, **MR**, magnetic resonance, **MU**, Monitor Unit, **OAR**, Organ at risk, **oART**, online adaptive radiotherapy, **PTV**, planning target volume, **QA**, Quality assurance, **SD**, standard deviation, **VMAT**, volumetric arc radiotherapy

## Abstract

**Background and purpose:**

The Ethos system has enabled online adaptive radiotherapy (oART) by implementing an automated treatment planning system (aTPS) for both intensity-modulated radiotherapy (IMRT) and volumetric modulated arc radiotherapy (VMAT) plan creation. The purpose of this study is to evaluate the quality of aTPS plans in the pelvic region.

**Material and Methods:**

Sixty patients with anal (n = 20), rectal (n = 20) or prostate (n = 20) cancer were retrospectively re-planned with the aTPS. Three IMRT (7-, 9- and 12-field) and two VMAT (2 and 3 arc) automatically generated plans (APs) were created per patient. The duration of the automated plan generation was registered. The best IMRT-AP and VMAT-AP for each patient were selected based on target coverage and dose to organs at risk (OARs). The AP quality was analyzed and compared to corresponding clinically accepted and manually generated VMAT plans (MPs) using several clinically relevant dose metrics. Calculation-based pre-treatment plan quality assurance (QA) was performed for all plans.

**Results:**

The median total duration to generate the five APs with the aTPS was 55 min, 39 min and 35 min for anal, prostate and rectal plans, respectively. The target coverage and the OAR sparing were equivalent for IMRT-APs and VMAT-MPs, while VMAT-Aps.

demonstrated lower target dose homogeneity and higher dose to some OARs. Both conformity and homogeneity index were equivalent (rectal) or better (anal and prostate) for IMRT-APs compared to VMAT-MPs. All plans passed the patient-specific QA tolerance limit.

**Conclusions:**

The aTPS generates plans comparable to MPs within a short time-frame which is highly relevant for oART treatments.

## Highlights

• Automated treatment planning system compared to manual planning.
• Equivalent plan quality between VMAT manually generated- and IMRT automatically generated plans.
• Evaluation of anal, prostate and rectum treatment plans.
• Generation of highly consistent IMRT automated plan within 2 to 3.5 min.

## Introduction

Radiotherapy treatment planning is a complex task. Inverse planning with intensity-modulated radiotherapy (IMRT) and volumetric modulated arc radiotherapy (VMAT) are currently used in most of the treatment planning systems (TPSs). Although the dose calculation itself is computer generated, IMRT and VMAT plan creation remains challenging due to many manual key steps and multiple iterations. To generate acceptable plans the dose defined for each tumor site and constraints for relevant organs at risk (OAR) needs to be manually translated into planning objectives assigned with weights that indicate their relative priority. Furthermore, substructures with different weighting often need to be created to precisely shape the dose distribution and optimize the treatment plan. These manual steps make the treatment planning process and the final treatment plan strongly dependent on the individual treatment planner’s experience and on department resources.

Replacing the manual steps in dose planning by automation could potentially reduce plan heterogeneity between treatment planners and departments. The goal is to automatically generate plans with a quality comparable to or better than those manually generated by an experienced planner. Over the past decade several such systems have been proposed, e.g. knowledge-based planning (KBP), multi-criteria optimization (MCO), template-based approaches and particle swarm optimization [1], [2], [3], [4], [5], [6], [7]. All of these tend to reduce the inter-planner variability, decrease the time spent on the optimization procedure and improve the plan quality. Daily online adaptive radiotherapy (oART) sets special demands on the performance of an automated TPS, i.e. accelerated beam re-optimization and dose calculation is needed as well as an ergonomic and fast treatment plan re-evaluation. Indeed, oART implementation must not considerably prolong the treatment process, as to avoid intra-fractional shifting and difficulty for the patient to tolerate a too long immobilization on the treatment couch [8]. An automated template-based TPS with this purpose has been recently developed and included in the Ethos platform (Varian Medical System (VMS), Palo Alto, Ca, USA) and clinically implemented at several institutions [9], [10], [11].

The aim of this study was to evaluate the plan quality achieved with Ethos TPS for anal, prostate and rectal cancer treatment, by comparing the automatically generated treatment plans (APs) with the manually generated and clinically accepted VMAT plans (MPs) created by experienced treatment planners with the Eclipse TPS (VMS, Palo Alto, CA, USA).

## Materials and Methods

### Patient and volume characterization

Three separate cohorts of each twenty consecutive patients with anal (n = 20), prostate (n = 20) or rectal (n = 20) cancer, that underwent curatively intended radiotherapy with VMAT at our department between December 2019 and February 2021, were retrospectively selected for this study. The median patient age was 70 years (range: 39–83 years). All patients underwent a simulation computed tomography (CT) scan in supine position, with a 2 mm slice thickness prior to the treatment. Magnetic Resonance (MR) images were acquired the same day as the simulation CT and co-registered to support accurate target volume delineation. All patients were instructed to have moderately filled bladder and to use rectal laxatives prior to the simulation scans. Target and OAR structures were manually delineated in Eclipse TPS and followed national guidelines for each tumor site [12], [13], [14]. For this study, the structures were imported into Ethos TPS for AP. The study was internally approved by our head of radiotherapy and was a part of our quality assurance (QA) procedure for implementing the clinical use of the AP in the Ethos TPS. Patient consent or ethical approval was not required.

For the patients with anal cancer, the primary clinical target volume (CTV-T) was derived from the gross tumor volume (GTV-T), separated into an upper and a lower part (intra-fractional motion difference), where an isotropic margin of 10 mm and of 15 mm was added to the upper and the lower part, respectively. The anal canal and/or rectum circumference was included in the CTV-T. The elective pre-sacral, ischiorectal, femoral, iliac, mesorectum and obturator regions were included in CTV-E for all patients. The anal canal was included in CTV-E for eleven patients, and the vagina was included for one patient. The planning target volume (PTV) for the primary target (PTV-T) and pelvic lymph nodes (PTV-E) were obtained by adding a 10 mm isotropic margin to the CTV-T and CTV-E, respectively. A total dose of 60 Gy and 48 Gy was delivered simultaneously to PTV-T and PTV-E, respectively, over 30 fractions, 5 fractions per week. Fourteen of the patients treated for anal cancer received simultaneous irradiation of positive lymph node(s) (CTV-N) to 60 Gy in 30 fractions. The CTV-N was obtained by adding a 5 mm isotropic margin to the GTV-N, and subsequently excluding muscles and bones. The PTV-N was defined as the CTV-N and a 10 mm isotropic margin. The main OARs were bladder, bowel bag and femoral heads.

The CTV-T for the patients with rectal cancer was defined as the union of the GTV-T and the rectum circumference at the tumor level with an additional 5 mm margin in the left–right and anterior-posterior directions and a 10 mm margin in the cranio-caudal direction. Bony structures were excluded from the CTV-T. An internal target volume (ITV-T) was created by expanding the CTV-T with 7 mm in the cranio-caudal and left–right directions, 4 mm in the posterior direction and 10 mm in the anterior direction. Bones and muscles were excluded from the ITV-T. The PTV-T was generated by applying a 5 mm isotropic margin to the ITV-T. The CTV-E included the entire mesorectum, the pre-sacral space and the lateral region including lymph nodes along the internal iliac vessels and the obturator nodes. A 5 mm margin in the anterior direction was added to the CTV-E to generate the ITV-E. Thereafter, the PTV-E was created as the ITV-E with a 5 mm additional isotropic margin. Both PTV-T and PTV-E were prescribed 50.4 Gy in 28 fractions, 5 fractions per week. The main OARs were bladder, bowel bag and femoral heads.

The CTV-T for patients with prostate cancer consisted of the prostate and the seminal vesicles (a GTV was not delineated). The CTV-E was delineated according to the guidelines of Lawton et al [15] and consisted of iliac nodes, obturator nodes and pre-sacral space. The PTV-T and PTV-E were obtained by adding a 5 mm isotropic margin except in cranio-caudal direction where 8 mm was used, to the CTV-T and CTV-E, respectively. PTV-T and PTV-E were simultaneously irradiated over 39 fractions, 5 times per week, to a total dose of 78 Gy and 56 Gy, respectively. The OARs were rectum, bladder, bowel bag and femoral heads.

### Treatment planning

The dose calculation algorithm used was Acuros XB (v.15.6.03, VMS) with heterogeneity correction and a calculation grid size of 2.5 mm for both TPS. The dose reporting mode was set as dose-to-medium. A single isocenter was used for all the patients except for two anal cancer patients where the target volume was longer than 28 cm and therefore the plans were optimized for two isocenters (with the same beam arrangement) separated by 8 cm in the cranio-caudal direction. All patients were treated with VMAT-MPs planned with the Eclipse TPS (v.15.6.05, VMS). Treatments were delivered on one of two linear accelerators (Linacs): a) TrueBeam (VMS, Palo Alto, CA, USA) with 6 MV photon beam and maximum dose rate of 600 Monitor Unit (MU)/min., or b) Halcyon (VMS, Palo Alto, CA, USA) Linac with closed bore design, 6 MV flattening filter free (FFF) photon beam and maximum dose rate of 800 MU/min. The TrueBeam is equipped with a Varian 120 multileaf collimator (MLC) (in each bank: 5 mm and 10 mm leaf width for the 40 central and the 20 outer leaves, respectively) [16]. The Halcyon is equipped with a double-stacked MLC (in each upper and lower bank 10 mm leaf width with 29 and 28 leaves, respectively) and have no secondary jaws [17].

**Fig. 1:**
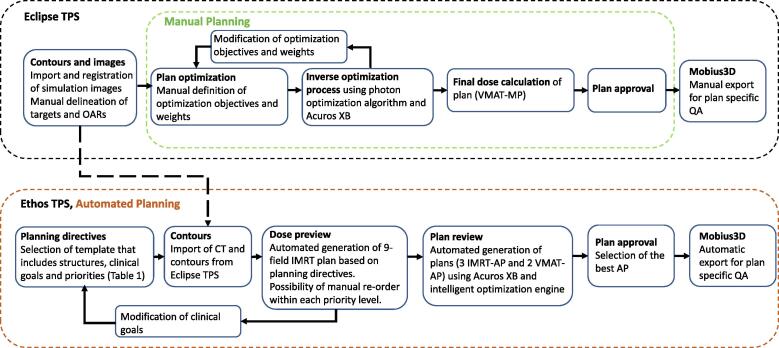
Schematic figure of the different steps of manually and automated plan generation. The VMAT-MPs were optimized by manually applying the dose-volume constraints to the CTV, PTV and OARs (Table 1) and a relative weight to each of them. Support structures, e.g. OAR minus PTV, ring around PTV and the normal tissue objective (NTO) tool in Eclipse, were utilized to shape the dose distribution and to reduce the dose outside the target. MUs were set to a maximum value of 400 MU to limit the plans complexity. The inverse optimization process was performed utilizing the photon optimization algorithm (v.15.6, VMS). The IMRT-AP and VMAT-AP were generated with Ethos TPS (v.1.0 MR1, VMS). Disease-specific treatment planning templates were optimized and defined based on five patient cases (including in each subgroups of this study) for each tumor site, i.e. anal, rectal and prostate cancer. Then, the final approved template was used to generate APs for all 20 patients in each site group.

A description of the MP and AP generation is included in Fig. 1. The VMAT-MPs correspond to the clinically approved plan used to treat each patient and were optimized by manually applying the dose-volume constraints to the CTV, PTV and OARs (Table 1) and a relative weight to each of them. The 60 patients treated with VMAT-MPs were re-planned with the Ethos TPS (v.1.0 MR1, VMS) which has previously been described by Archambault et al [18]. To summarize, an algorithm, named intelligent optimization engine, generates a dose distribution based on the clinical goals in a template set up by the user. Depending on the internal configuration, up to three IMRT-APs (7-, 9- and 12-field) and two VMAT-APs (2- and 3-arcs) are automatically generated. By deselecting the calculation of one of the plan types, the AP generation duration for one patient is decreased. For this study the maximum (5) plans were created for each patient. The IMRT-AP and VMAT-AP that best fulfilled the clinical goals (Table 1) and the visual inspection criteria for dose distribution, e.g. no excess dose in the healthy tissue or underdosage in the CTV, were selected by an experienced medical physicist (LC), exported and imported into Eclipse TPS for further evaluation.

**Table 1:** Summary of structure names, corresponding clinical goals and objectives used in the template for the APs, and the achieved values for MPs and APs for the treatment of anal, prostate and rectal cancer. The p values are extracted from comparisons between MPs and APs.

	**Priority**	**Structure Name**	**Clinical Goals**	**Objective AP**	**Achieved value VMAT-MP Median (IQR)**	**Achieved value IMRT-AP Median (IQR)**	**p value**	**Achieved value VMAT-AP Median (IQR)**	**p value**
**Anal Cancer**	1	**CTV-T/N**	V_95%_ = 100 %	V_97%_ ≥ 100%	100	100		100	
	1	**CTV-E_tot**	V_95%_ = 100 %	V_97%_ ≥ 100%	100	100		100	
	1	**PTV-T**	V_95%_ ≥ 99 % V_90%_ = 100 % V_105%_ ≤ 1 %	V_95%_ ≥ 99% V_90%_ ≥ 100% D1cc ≤ 105%	99.5 (99.2–99.9) 100 0	99.8 (99.7–99.9) 100 0	0.126	99.7 (99.3–99.8) 100 0	0.935
	1	**PTV-N**	V_95%_ ≥ 99 % V_90%_ = 100 % V_105%_ ≤ 1 %	V_95%_ ≥ 99% V_90%_ ≥ 100% D1cc ≤ 105%	99.6 (99.0–99.7) 100 0	99.8 (98.4–99.8) 100 0	0.597	99.4 (97.6–99.9) 100 0.1 (0–0.4)	0.765 0.003
	1	**PTV-E**	V_95%_≥ 98 % V_90%_ = 100 %	V_95%_ ≥ 98 % V_90%_ ≥ 100 %	99 (98.5–99.3) 100	99.9 (99.8–99.9) 100	<10^-5^	99.7 (99.5–99.9) 100	0.002
	1	**PTV-EminusPTV-T**	V_107%_ ≤ 3 %	V_107%_≤ 1%	1.9 (1.0–2.5)	0.3 (0.1–0.7)	<10–4	7.6 (4.3–20.9)	<10^-3^
	2	**Bowel bag**	V_30Gy_ ≤ 600 cc V_45Gy_ ≤ 300 cc	V_30Gy_ ≤ 600 cc V_45Gy_ ≤ 300 cc	611.6 (537.7–768.3) 331.6 (285.7–447.6)	553.2 (509.1–634.9) 332.6 (278.1–406.7)	0.164 0.635	636.5 (572.6–734.5) 362.6 (307.1–437.9)	0.840 0.620
	2	**Bladder**	V_50Gy_ ≤ 20 % V_35Gy_ ≤ 75 %	V_50Gy_ ≤ 20 % V_35Gy_ ≤ 75 %	5.7 (0.8–15.5) 71.8 (63.3–85.0)	4.4 (0.8–10.6) 67.7 (53.9–71.9)	0.645 0.086	7.1 (1.6–16) 84.2 (75.2–92.2)	0.516 0.025
	2	**Femoral Head**	D_max_ ≤ 52 Gy	D_max_ ≤ 52 Gy	46.1 (44.8–48.5)	45.5 (43.7–47.9)	0.176	47.5 (45.5–50.5)	0.162

**Rectal Cancer**	1	**CTV-tot**	V_95%_ ≥ 100 %	V_98%_ ≥ 100 %	100	100		100	
	1	**PTV-tot**	V_95%_ ≥ 99 % V_105%_ ≤ 1 %	V_95%_ ≥ 99 % D_1cc_ ≤ 103%	99.6 (99.3–99.8) 0	99.9 (99.9–100.0) 0	< 10^-5^	99.3 (98.9–99.6) 0.45 (0.1–2.9)	0.044 <10^-6^
			V_90%_ ≥ 100 %	V_90%_ ≥ 100 %	100	100		100	
	2	**Bladder**	V_50Gy_ ≤ 20 % V_35Gy_ ≤ 75 %	V_50Gy_ ≤ 20 % V_35Gy_ ≤ 75 %	5.6 (3.1–10.1) 39.8 (29.8–47.5)	3.2 (1.1–4.4) 44.0 (34.4–53.8)	0.011 0.267	3.1 (0.7–4.9) 41 (31.1–51.5)	0.019 0.675
	2	**Bowel bag**	V_45Gy_ ≤ 300 cc V_30Gy_ ≤ 600 cc	V_45Gy_ ≤ 300 cc V_30Gy_ ≤ 600 cc	256.9 (226.7–316.1) 431.7 (356.2–536.2)	256.5 (220.1–335.5) 482.3 (399.8–564.3)	0.850 0.218	243.8 (208.3–324.8) 420.2 (370.9–565.3)	0.579 0.665
	2	**Femoral head**	D_max_ ≤ 52 Gy	D_max_ ≤ 52 Gy	38.2 (36.1–40.5)	33.8 (28.5–39.7)	0.504	36.7 (31.7–38.7)	0.970

**Prostate Cancer**	1	**CTV-T**		D_98%_ ≥ 76.5 Gy	77.1 (76.8–77.4)	78.0 (77.8–78.1)	<10^-6^	77.6 (77.4–77.7)	<10^-4^
	1	**CTV-E**		D_98%_ > 55 Gy	55.2 (54.7–55.5)	56.2 (56.0–56.3)	<10^-6^	55.9 (55.8–56.1)	<10^-4^
	1	**PTV-T**	V_95%_ ≥ 95 % D_max_ ≤ 107%	V_97%_ ≥ 95% D_1cc_ ≤ 105%	98.7 (98.3–99.1) 0	98.2 (97.5–98.6) 0	0.019	99.0 (98.6–99.2) 0	0.218
	1	**PTV-E** **PTV-EminusPTV-T**	V_95%_ ≥ 95 % V_107%_ ≤ 3%	V_96%_ ≥ 95% V_105%_ ≤ 5%	98.7 (97.9–99.4) 3.4 (2.4–6.5)	98.9 (98.7–99.2) 3.6 (2.6–4.6)	0.273 0.636	99.6 (99.3–99.8) 6.1 (4.6–7.4)	<10^-3^ 0.016
	2	**Rectum**	D_1cc_ ≤ 78 Gy V_70Gy_ ≤ 10 cc V_60Gy_ ≤ 30 % V_40Gy_ ≤ 60 %	D_1cc_ ≤ 77 Gy V_70Gy_ ≤ 10 cc V_60Gy_ ≤ 30 % V_40Gy_ ≤ 60 %	76.2 (74.5–77.0) 4.5 (3.0–7.0) 42.7 (33.1–50.2) 15.5 (12.2–19.0)	76.9 (75.5–77.8) 5.2 (3.5–7.9) 53.6 (48.8–54.8) 16.5 (13.7–20.0)	0.218 0.394 0.402 0.013	77.1 (76.0–78.4) 5.2 (4.2–8.0) 53.0 (51.6–56.5) 17.6 (14.6–21.0)	0.076 0.189 0.140 0.003
	2	**Bowel bag**	V_45Gy_ ≤ 300 cc V_30Gy_ ≤ 600 cc	V_45Gy_ ≤ 300 cc V_30Gy_ ≤ 600 cc	383.8 (315.5–449.1) 591.2 (499.7–644.7)	377.5 (292.2–407.0) 577.0 (553.7–660.6)	0.410 0.394	397.3 (335.2–430.0) 635.8 (551.6–733.2)	0.830 0.239
	2	**Femoral head**	D_max_ ≤ 52 Gy	D_max_ ≤ 52 Gy	47.3 (46.3–48.9)	42.0 (39.0–44.8)	< 10^-6^	42.1 (37.7–44.1)	< 10^-7^
	2	**Bladder**	D_mean_ ≤ 62 Gy	D_mean_ ≤ 62 Gy	49.1 (47.8–51.7)	47.8 (45.2–49.1)	0.072	48.9 (46.1–50.5)	0.507

### Plan specific quality assurance

Calculation-based pre-treatment plan QA was conducted for all MPs and APs using Mobius3D (Mobius Medical Systems, LP, Houston, TX) independent dose calculation software and global gamma analysis (tolerance: 3%/3mm, 10% threshold).

### Plan comparison

The dose distributions were evaluated through assessment of relevant metrics derived from the dose volume histogram (DVH) for the PTV and OARs. The dose conformity to the shape and the size of the PTV-T and PTV-N was evaluated with the conformity number (CN) [19], where a value close to one indicated an acceptable plan quality. The homogeneity of the dose distribution within the PTV-T, PTV-N and PTV-E were represented by the homogeneity index (HI) [20], where a value close to 0 indicated a homogenous plan. The two indices were defined as:.

(1) $$\mathrm{HI}=\frac{D_{2\%}-D_{98\%}}{D_{50\%}}$$

(2) $$\mathrm{CN}=\frac{V_{95\%,PTV-HD}^{2}}{V_{\mathrm{PTV}-HD}\times V_{95\%,Body}}$$

where D_x%_ is the minimum dose received by the hottest x% of the PTV volume, i.e. D_2%_ is the near maximum dose, D_98%_ is the near minimum dose, and D_50%_ is the median dose to the PTV. *V*_PTV-HD_ is the volume of the PTV high dose (HD), i.e. the PTV-T for the rectum and prostate plans and the union of PTV-T and PTV-N for the anal plans. *V*_95%, PTV-HD_ and *V*_95%, Body_ are the volumes receiving 95% or more of the prescribed dose for the PTV-HD and body, respectively. For each patient the DVHs were extracted from the Eclipse TPS with a volume resolution of 0.1% and a dose resolution of 0.1%. The differences (ΔDVH, in percentage points) calculated as the AP minus the MP were computed in Matlab for targets and OARs. For each dose the median and interquartile range (IQR) of the corresponding volume differences were calculated. Furthermore, the modulation factor (MU/Gy) and the median treatment planning time for APs were recorded.

### Statistical analyses

Statistical evaluation of the extracted parameters was performed in Matlab (version R2019a, The MathWorks, Natick, Massachusetts, USA). The differences among the groups of plans were assessed using non-parametric tests (Wilcoxon signed-rank test). All the tests were not fully independent and therefore no correction for multiple testing has been applied. However, all p values are provided and thus the impact of a simple Bonferroni correction can easily be estimated, i.e. if the overall alpha is set to 0.05 the alpha for each test would be 0.05 divided by the number of tests.

## Results

### Treatment planning

A total of 300 plans were automatically created for the patients using the Ethos TPS. The 12-field, 9-field and 7-field IMRT-APs were considered and selected as the best IMRT-AP for 57% (n = 34), 35% (n = 21) and 8% (n = 5, only rectum) of the patients, respectively. For all VMAT-APs, except for three rectal patients, the 3-arc plans were better at fulfilling the constraints compared to the 2-arc plans. The median duration to generate the 12-field IMRT-APs was 4.1 min, 3.0 min and 3.1 min for anal, rectal and prostate plans respectively (Table 2) while it took approximately 4 times longer to generate the VMAT-APs. A larger amount of MU/Gy was observed for APs compared to MPs (Table 3).

**Table 2:** Treatment planning duration of APs: median (IQR) of preparation, optimization and calculation (generation), and total duration for anal, rectum and prostate cancer.

	**Anal**	**Rectum**	**Prostate**
**Median (IQR) preparation duration [min]**	15.0 (14.0–16.0)	8.5 (7.8–10)	7.0 (5.0–10.3)
**Median (IQR) generation of IMRT 7 [min]**	3.3 (3.0–3.8)	2.2 (2.1–2.5)	2.4 (2.3–2.5)
**Median (IQR) generation of IMRT 9 [min]**	3.9 (3.4–4.2)	2.6 (2.4–2.8)	2.8 (2.7–3.0)
**Median (IQR) generation of IMRT 12 [min]**	4.1 (3.8–4.9)	3.0 (2.7–3.2)	3.1 (3.0–3.4)
**Median (IQR) generation of VMAT 2 [min]**	17.5 (15.8–18.2)	10.1 (9.5–10.9)	12.0 (11.7–13.4)
**Median (IQR) generation of VMAT 3 [min]**	18.1 (15.7–18.6)	11.2 (9.9–11.8)	12.7 (12.1–14.1)
**Median (IQR) total duration [min]**	54.7 (49.8–62.6)	35.2 (29.8–38.7)	36.8 (33.3–41.4)

**Table 3:** Median (IQR) value of CN, HI, the modulation factor (MU/Gy) and the Mobius3D gamma passing rate for VMAT-MPs, the selected IMRT-APs and VMAT-APs calculated for anal, prostate and rectal cancer patient. The p values are calculated between APs and MPs.

			**CN**	**HI PTV-T**	**HI PTV-N**	**HI PTV-E**	**Modulation factor (MU/Gy)**	**Gamma passing rate (3%/3mm)**
**Anal**	**VMAT-MP**	**Median (IQR)**	0.88 (0.86–0.91)	0.06 (0.05–0.06)	0.06 (0.05–0.07)	0.11 (0.09–0.12)	287.3 (225.9–315.4)	96.7% (94.5%-97.5%)
	**IMRT-AP**	**Median (IQR)**	0.94 (0.93–0.97)	0.06 (0.06–0.06)	0.06 (0.06–0.07)	0.07 (0.07–0.08)	1145.0 (1076–1263)	98.1% (97.5%-98.8%)
		**p value**	≤10^-4^	0.004	0.476	≤10^-5^		0.001
	**VMAT-AP**	**Median (IQR)**	0.90 (0.88–0.93)	0.06 (0.06–0.07)	0.08 (0.06–0.09)	0.12 (0.10–0.15)	335.7 (317.9–364.6)	96.1% (95.6%-98.1%)
		**p value**	0.148	0.003	0.033	0.107	≤10^-3^	0.694

**Rectum**	**VMAT-MP**	**Median (IQR)**	0.84 (0.82–0.84)	0.05 (0.05–0.06)			323.3 (284.8–339.2)	95.7% (94.3%-98.7%)
	**IMRT-AP**	**Median (IQR)**	0.87 (0.85–0.88)	0.04 (0.04–0.05)			801.2 (789.4–961.0)	99.7% (99.6%-99.8%)
		**p value**	≤ 10^-4^	≤ 10^-3^				< 10^-5^
	**VMAT-AP**	**Median (IQR)**	0.91 (0.91–0.92)	0.08 (0.07–0.10)			418.0 (380.6–444.9)	98.2% (97.8%-98.8%)
		**p value**	≤ 10^-7^	≤10^-6^			≤10^-6^	0.083

**Prostate**	**VMAT-MP**	**Median (IQR)**	0.84 (0.82–0.86)	0.07 (0.06–0.07)		0.19 (0.13–0.36)	271.3 (252.7–311.1)	97.6% (97.1%-98.7%)
	**IMRT-AP**	**Median (IQR)**	0.91 (0.90–0.92)	0.07 (0.06–0.07)		0.20 (0.14–0.30)	890.4 (749.4–999.7)	99.9% (99.8%-100%)
		**p value**	≤ 10^-6^	0.250		0.839		<10^-6^
	**VMAT-AP**	**Median (IQR)**	0.90 (0.89–0.91)	0.06 (0.06–0.22)		0.27 (0.22–0.35)	406.1 (387.7–459.0)	99.4% (99.1%-99.6%)
		**p value**	≤10^-6^	0.041		0.262	≤10^-6^	< 10^-4^

### Plan specific quality assurance

All APs fulfilled the tolerance with a global gamma passing rate (global 3%/3mm, 10% threshold) above 95%, and the clinical MPs were all approved for the clinical treatment.

### Plan comparison

**Fig. 2:**
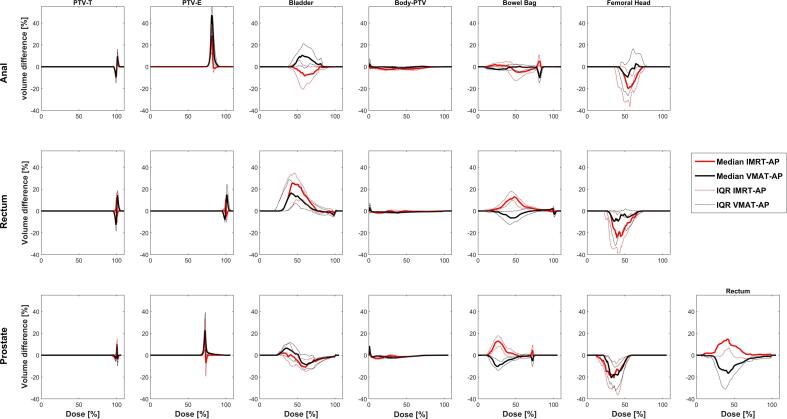
Median of the dose volume histogram difference (ΔDVH) between IMRT-AP minus VMAT-MP (red solid line) and between VMAT-AP minus VMAT-MP (black solid line) and the IQR (dotted lines) for the cohort of anal, rectum, and prostate patients. Data are shown for all the PTV volumes and the OARs. Note that the plots have different axes scaling for the PTV-E volume. (For interpretation of the references to colour in this figure legend, the reader is referred to the web version of this article.)

PTV coverage and healthy tissue (Body-PTV) sparing were similar between MPs and APs (Fig. 2). For anal plans, IMRT-AP ΔDVHs indicated better OAR sparing. For rectum plans, the VMAT-MPs presented a better sparing of bladder compared to both APs and for bowel bag compared to IMRT-AP but not compared to VMAT-AP. The APs, especially IMRT-APs, indicated lower dose to the femoral heads. For prostate plans, the VMAT-MP ΔDVHs displayed lower dose to both the rectum and the bowel bag compared to IMRT-AP while the opposite was true compared to VMAT-AP. For the bladder the results are mixed, while the APs were better at reducing the dose to the femoral heads.

For the CN a difference was observed to be in favor of the APs compared to MPs for the three disease sites (p ≤ 10^-4^ except for VMAT-APs anal) (Table 3). The HI was similar between the plans and in general very low.

**Fig. 3:**
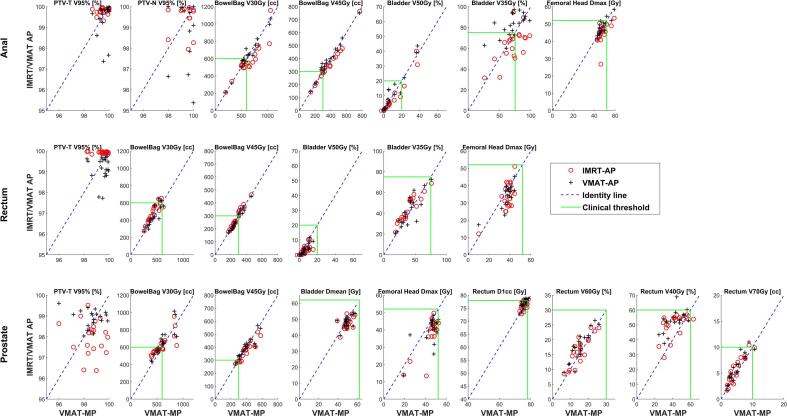
Doses to PTV and OARs for IMRT-APs (red circle) and VMAT-APs (black cross) as function of VMAT-MPs (x-axis); the blue line indicates the identity line, while the green line indicates our clinical threshold for each metric as define in Table 1. Note that the plots have different axes scaling. (For interpretation of the references to colour in this figure legend, the reader is referred to the web version of this article.)

For anal patients, the target coverage (V_95%_ and V_105%_ of the PTV-T and PTV-N) was almost identical between the plans (Fig. 3, Table 1). The V_107%_ of PTV-E was higher and not fulfilling the clinical goal for most VMAT-AP compared to VMAT-MPs (p ≤ 10^-3^), while the value was a slightly lower for IMRT-APs compared to VMAT-MPs (p ≤ 10^-4^). Similarly, the bladder V_35Gy_ was higher and not fulfilling the clinical goal for most VMAT-APs compared to VMAT-MPs (p = 0.025), while there was a small tendency for better IMRT-APs compared to VMAT-MPs measure (p = 0.086) (Table 3, Fig. 2).

For the rectal patients, there was a small increase in the PTV coverage with IMRT-AP (p ≤ 10^-6^) compared to MPs, while VMAT-APs presented the lowest V_95%_ PTV coverage (p = 0.044) and a higher V_105%_ value (p ≤ 10^-5^) (Fig. 3, Table 1). Both IMRT-AP and VMAT-AP were better at sparing the bladder with lower V_50Gy_ (p < 0.02).

For prostate cases differences were small, but VMAT-MPs demonstrated a slightly better (V_40Gy_, p < 0.01) or equivalent sparing of the rectum compare to both APs, while the maximal dose received by the femoral head was lower with the IMRT-AP compared to the VMAT-MPs (p 〈10^−6^) (Fig. 3, Table 1).

## Discussion

This study explored the potential of IMRT-AP and VMAT-AP implemented in the Ethos TPS (v.1.0 MR1) for pelvic treatments. The main purpose of this investigation was to verify the hypothesis that the Ethos system can achieve plan quality equivalent to or better than our clinical practice with manual treatment planning. The three disease sites, i.e. anal, rectum, and prostate, were selected due to the potential benefit of oART.

The data indicate that the IMRT-APs result in equivalent plan quality to VMAT-MPs in terms of target coverage and OAR sparing, while the VMAP-APs had lower dose homogeneity in the target volume and higher dose to some OAR (Fig. 3, Table 1). These results are in line with the observed benefits of auto-planning in a previous study [21]. Based on this study we do no longer generate the VMAT-APs in our clinical practice (in order to save time and since the IMRT-APs are always better or equal to the VMAT-APs).

Regarding planning and treatment efficiency, VMAT-APs resulted in MU increase for all disease sites, which is in agreement with another auto-planning engine [3] and suggests an increase of plan complexity and fluency modulation. However, the pre-treatment verification with Mobius3D demonstrates similar (rectum plans) or higher (prostate and anal plans, p 〈10^−4^) gamma passing rates for both IMRT and VMAP-APs (Table 3). Furthermore, the MUs of VMAT-MP were set to a fixed maximum value in our practice.

Median overall planning time (Table 2) including human inputs, optimization loop processes and calculation times was 55 min, 39 min and 35 min for anal, prostate and rectal APs respectively for five plans (3 IMRT and 2 VMAT plans), which is similar to the time needed to generate one VMAT-APs with the auto-planning used by Cilla et al [3] and approximately four times shorter than VMAT-MP generation in their study. Based on timing measurements of an experienced planner in our clinic, we estimate the time to generate one VMAT-MP to 60 to 120 min. However, this time depends on the experience of the planner and could be further reduced using e.g. scripts or templates, not applied in this study.

In our study, only a small set of training patients (five) for each anatomical site was necessary as starting point to create templates that generate plans of high quality, while for KBP models much more patient data are needed [22]. Another advantage of template-based planning is to reduce the inter- and intra-planner variations, to ensure higher homogeneity in plan quality [21] and to push the OAR sparing beyond the objectives [3] like it is the case in our study for the femoral head and the bladder, for the prostate and anal cases, respectively (Fig. 2). Several studies have demonstrated treatment time reductions with the closed bore design of Halcyon machines compared to C-arm Linacs [23], [24] with potential reduction of intra-fraction motion. In our experience, the most modulated 12-field IMRT plans for anal cancer delivered on a Halcyon has a delivery time similar to that of a 3-arc VMAT on a Truebeam.

A potential weakness of this study is that the MPs quality should be as high as possible to avoid favor to APs. In our case all clinically MPs were created by experienced treatment planners in a clinical context. In our study we do not compare the performance of TrueBeam to Halcyon, this has been done by other investigators [25].

In conclusion, this study demonstrated the capability of Ethos TPS (v 1.0 MR1) to generate highly consistent IMRT-APs in the pelvic region with equivalent target coverage and OAR sparing, compared to VMAT-MPs. The quality of VMAT-APs, however, needs to be improved. For optimal oART Ethos plan quality, where a time-efficient procedure to calculate and re-optimize treatment plans is essential, the 12-field IMRT-AP should be used for anal and large prostate target volumes, while a 9-field IMRT-AP is preferable for small prostate and rectal target volumes.

Funding.

The Radiotherapy Research Unit, Department of Oncology receives funding from Varian, ViewRay and BrainLab for several projects outside the submitted work.

## Declaration of Competing Interest

The authors declare that they have no known competing financial interests or personal relationships that could have appeared to influence the work reported in this paper.
